# Supplementary material for: Identification of photoperiod-induced specific miRNAs in the adrenal glands of Sunite sheep (Ovis aries)
Source: Front Vet Sci. 2022 Jul 22;9:888207. doi: 10.3389/fvets.2022.888207 (PMC9354845; doi:10.3389/fvets.2022.888207)
Supplement: Supplementary file 1 [file Data_Sheet_1.ZIP › Supplementary file/miRDeep2 file.zip!/miRDeep2 file/Know_Structure/LP42A1/oar-mir-21.pdf]

miRBase precursor : oar-mir-21
Total read count : 216458
oar-miR-21 read count : 216442
remaining reads : 16

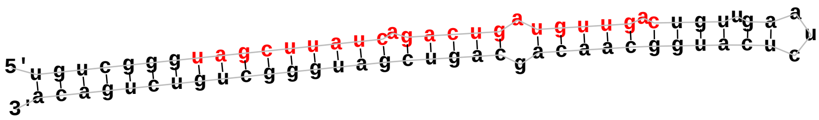


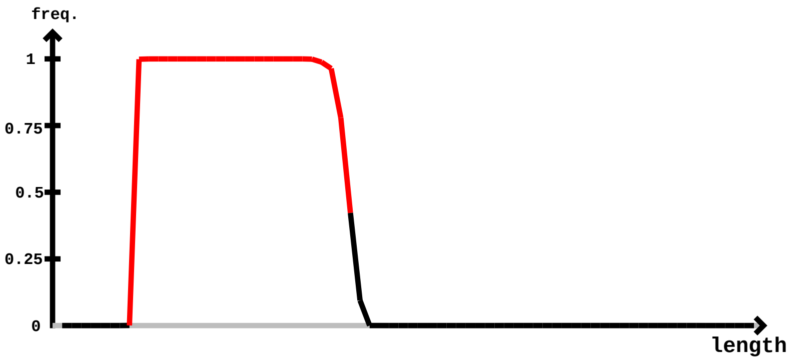


oar-miR-21

5'- ugucggg**uagcuuaucagacugauguugac**uguugaaucucauggcaacagcagucgaugggcugucugaca -3' exp

| ((((((((((((((((.(((((.(((((.((((.((...)))))))))))).)))))))))))))))))))) | reads | mm | sample |
|---|---|---|---|
| ....ggguagcuuaucagacugauguu............................................. | 1 | 0 | seq |
| ......guagcuuaucagacugaugu.............................................. | 2 | 0 | seq |
| ......guagcuuaucagacugauguu............................................. | 6 | 0 | seq |
| ......guagcuuaucagacugauguug............................................ | 61 | 0 | seq |
| ......guagcuuaucagacugauguuga........................................... | 29 | 0 | seq |
| ......guagcuuaucagacugauguugac.......................................... | 10 | 0 | seq |
| ......guagcuuaucagacugauguugacu......................................... | 4 | 0 | seq |
| .......uagcuuaucagacugau................................................ | 10 | 0 | seq |
| .......uagcuuaucagacugaug............................................... | 190 | 0 | seq |
| .......uagcuuaucagacugaugu.............................................. | 2420 | 0 | seq |
| .......uagcuuaucagacugauguu............................................. | 5131 | 0 | seq |
| .......uagcuuaucagacugauguug............................................ | 39866 | 0 | seq |
| .......uagcuuaucagacugauguuga........................................... | 77182 | 0 | seq |
| .......uagcuuaucagacugauguugac.......................................... | 70885 | 0 | seq |
| .......uagcuuaucagacugauguugacu......................................... | 20351 | 0 | seq |
| .......uagcuuaucagacugauguugacug........................................ | 5 | 0 | seq |
| .......uagcuuaucagacugauguugacugu....................................... | 13 | 0 | seq |
| .......uagcuuaucagacugauguugacuguu...................................... | 3 | 0 | seq |
| .......uagcuuaucagacugauguugacuguug..................................... | 1 | 0 | seq |
| .......uagcuuaucagacugauguugacuguugaau.................................. | 1 | 0 | seq |
| ........agcuuaucagacugaugu.............................................. | 2 | 0 | seq |
| ........agcuuaucagacugauguu............................................. | 7 | 0 | seq |
| ........agcuuaucagacugauguug............................................ | 35 | 0 | seq |
| ........agcuuaucagacugauguuga........................................... | 70 | 0 | seq |
| ........agcuuaucagacugauguugac.......................................... | 81 | 0 | seq |
| ........agcuuaucagacugauguugacu......................................... | 45 | 0 | seq |
| .........gcuuaucagacugaugG.............................................. | 1 | 1 | seq |
| .........gcuuaucagacugauguug............................................ | 3 | 0 | seq |
| .........gcuuaucagacugauguuga........................................... | 11 | 0 | seq |
| .........gcuuaucagacugauguugac.......................................... | 12 | 0 | seq |
| ..........cuuaucagacugauguugac.......................................... | 1 | 0 | seq |
| ...........uuaucagacugauguug............................................ | 1 | 0 | seq |
| ...........uuaucagacugauguuga........................................... | 2 | 0 | seq |
| ...........uuaucagacugauguugac.......................................... | 1 | 0 | seq |
| ............uaucagacugauguugac.......................................... | 1 | 0 | seq |

**oar-miR-21**

```
ugucggguagcuuaucagacugauguugacuguugaaucucauggcaacagcagucgaugggcugucugaca
...............cagacugauguugacuguugaauc.................................    1    0    seq
.............................................caacagcagucgaugggcugu......    4    0    seq
.............................................caacagcagucgaugggcuguc.....    8    0    seq
...............................................acagcagucgaugggcugucu....    1    0    seq
```
